# Does social distancing affect the processing of brand logos?

**DOI:** 10.1002/brb3.2501

**Published:** 2022-02-25

**Authors:** Stefania D'Ascenzo, Elisa Scerrati, Caterina Villani, Renata Galatolo, Luisa Lugli, Roberto Nicoletti

**Affiliations:** ^1^ Department of Philosophy and Communication University of Bologna Bologna Italy; ^2^ Department of Biomedical, Metabolic and Neuroscience University of Modena and Reggio Emilia Reggio Emilia Italy

**Keywords:** brand logos, *distancing effect*, perception, social distancing, spaced

## Abstract

Social distancing and isolation have been imposed to contrast the spread of COVID‐19. The present study investigates whether social distancing affects our cognitive system, in particular the processing of different types of brand logos in different moments of the pandemic spread in Italy. In a size discrimination task, six different logos belonging to three categories (letters, symbols, and social images) were presented in their original format and spaced. Two samples of participants were tested: one just after the pandemic spread in Italy, the other one after 6 months. Results showed an overall *distancing effect* (i.e., spaced stimuli are processed slower than original ones) that interacted with the sample, revealing a significant effect only for participants belonging to the second sample. However, both groups showed a *distancing effect* modulated by the type of logo as it only emerged for social images. Results suggest that social distancing behaviors have been integrated in our cognitive system as they appear to affect our perception of distance when social images are involved.

## Graphical abstract

In this manuscript, we investigate the possibility that the social distancing imposed to contrast the spread of COVID‐19 affected our cognitive system. We focused on the processing of commercial logos presented originally and spaced. Results showed that the social distancing behaviors imposed have been integrated in our cognitive system since it affects our perception of distancing processing when social images are involved.

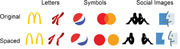

## 1 INTRODUCTION

All over the world, states and local governments introduced social distancing policies and isolation in response to the coronavirus (COVID‐19) pandemic that started in 2020. The adoption of such behaviors has drastically changed our daily life and the social and interactional contexts we are involved in. Since then, several studies have been conducted to investigate the impact of social distancing on a psychological level (see for example, Triberti et al., 2021; Wei, 2020), and its influence on mental health and cognitive abilities (see Maggi et al., 2021; Santangelo et al., 2021). For example, research has focused on how distancing and social isolation affected people on the basis of their socioeconomic condition (e.g., Williams et al., 2020) and on how the pandemic affected our cognition and wellbeing (e.g., De Pue et al., 2021). Regarding mental health, Maggi et al. (2021) report a longitudinal study on the elderly population, showing that the restrictive measures contribute to the rise of psychological symptoms such as depression, anxiety, anger, and subjective cognitive failures.

On a cognitive level, researches have been focused on the impact of the psychological symptoms on the occurrence of cognitive failures, defined as subjective perceptions of lapses in cognition. In particular, Santangelo et al. (2021) showed that about 30% of participants reported cognitive failures (i.e., attentive and memory difficulties) due to increased depression and anger during quarantine. The authors concluded that resilience could represent a factor to reduce the impact of depression and anger on the development of subjective cognitive failures.

The pandemic context is mostly characterized by social distancing and by wearing the surgical mask: both behaviors have been reported to influence our cognitive abilities, the perception of interpersonal distance and social interactions. For instance, Cartaud et al. (2020) investigated how wearing a facemask affects social distancing: participants reported a significant decrease in preferred interpersonal distance when the social interaction involved face stimuli wearing a facemask compared to face stimuli with no mask. Xu and Cheng (2021) observed that participants who were more risk averse practiced more social distancing and mask‐wearing.

More generally, the influence of context on our cognitive processes has been well‐documented in experimental psychology. For instance, in the Flanker task (Eriksen & Eriksen, 1974; Lappin et al., 2021 for recent discussion) participants are instructed to identify a relevant characteristic of the stimulus (e.g., the orientation of an arrow stimulus) that is presented in a context that can be congruent or incongruent with it. Thus, participants may need to identify an arrow pointing to the left among other arrows pointing to the left (congruent context), or among other arrows pointing to the right (incongruent context). Results demonstrate that participants are influenced by the context surrounding the target arrow as slower response times are observed with the incongruent compared to the congruent context, despite the fact that the latter is irrelevant to the task. Furthermore, research on memory highlighted the so‐called priming effect, that is, the influence that the introduction of one stimulus (often a word or an image) has on how people respond to a subsequent stimulus (e.g., Collins & Loftus, 1975; Meyer & Schvaneveldt, 1971). For example, the word *nurse* is recognized faster following the word *doctor* than the word *bread*. More generally, priming occurs whenever exposure to one thing affects an individual's subsequent behavior or thoughts. Moreover, in psycholinguistic research the importance of the context has been highlighted by the word superiority effect (WSE, Reicher, 1969; Wheeler, 1970) whereby letters are identified better when embedded within a word than in a nonword string, or when presented alone.

For the purposes of our study, it is also worth emphasizing that stimuli can be processed faster on the basis of their familiarity (e.g., Wang et al., 1994). In a visual search task, Wang et al. (1994) observed that when low‐level features of targets and distractors were held constant, performance was affected by familiarity. Specifically, they obtained faster reaction times in a visual search with familiar stimuli (i.e., a single upright letter) compared to unfamiliar ones (i.e., a single inverted letter). Importantly, our object recognition system automatically connects perceived stimuli with stored knowledge (Grill‐Spector & Kanwisher, 2005; Thorpe et al., 1996), leading to a faster recognition of stimuli we already know since semantic information is already available. In addition, there is evidence that we are able to detect an object faster when we are familiar with it (Konkle & Oliva, 2012).

On the basis of the above reported evidence highlighting the impact of COVID on our cognitive processes and social behaviors, the influence that context exerts on these processes, and how we typically react to familiar stimuli, we investigated how the current pandemic context impacts on the processing of original and spaced brand logos. This choice was motivated by an advertising campaign of some brands (e.g., Macdonald's, Coca‐Cola), which have modified their logos to promote social distancing during the pandemic.

Logos as visual cues help firms communicate their identities and capture consumers’ attention (Peracchio & Meyers‐Levy, 2005). Given their importance, several studies have investigated the role of logo representation and, in particular, how the characteristics of logos can influence the perception of the properties of a product or a company. For example, it has been demonstrated that preference judgments are influenced by the circularity or angularity of a logo (Jiang et al., 2016), its typographic character (Grohmann et al., 2013), its asymmetry (Luffarelli et al., 2019) and its orientation (Zhong et al., 2018; for a review see Kim & Lim, 2019). In addition, Philiastides and Ratcliff (2013) showed that logos and brands can have a great influence on decision‐making processes as they can affect our choices if present on a specific clothing.

Given the role of logos in orienting consumers' behaviors, it could be relevant to investigate whether and to what extent their perception might be affected by social contextual constraints, such as those imposed by social distancing.

In the present study, we used a size discrimination task where participants had to determine whether the stimulus was large or small. Original and spaced logos belonging to three different categories (letters, symbols, and social images) were used as targets. We expected to observe a *distancing effect*, whereby spaced logos, that is, unfamiliar logos, were processed slower than original ones, i.e., familiar logos, in line with previous evidence (Qin et al., 2014). Qin et al. (2014) indeed reported that in a visual search task familiar logos were found faster than unfamiliar ones. Moreover, we expected to observe that, within spaced logos, social images logos, evoking the practice of social distancing, might be processed slower than letters and symbols spaced logos because they are associated with forced and unnatural behavior. This could result in a greater cognitive effort to elaborate them, which might be reflected by slower RT. Therefore, we predict a modulation of the *distancing effect* with respect to the different kinds of logo. More specifically, we hypothesize to find a greater *distancing effect* for social logo images as these images depict human‐like figures, thus recalling an unnatural/unfamiliar social interaction such as the one imposed by anti‐COVID measures. After all, social images recall a social dimension that is the aspect of our daily life most affected by the adopted behavior during the pandemic. In addition, we predict that the more one has been exposed to these measures, the greater the effect. To test this prediction, two different samples were tested: the first one just after the pandemic spread started in Italy and the population began acting following distancing practices (June to July 2020), and the second one after 6 months (January 2021), when the distancing behaviors were already well established. The *distancing effect* should be larger for participants who were tested in January 2021 compared to the sample tested in June/July 2020.

## 2 METHODS

### 2.1 Participants

We calculated the sample size required to achieve 80% power to detect a significant *Logos* (MacDonald's, Kellogg's, Mastercard, Pepsi, Finder, Robe di Kappa) × *Distancing* (spaced, original) interaction with the MorePower software (i.e., Campbell & Thompson, 2012). With an effect size *f* = 0.387 (Cohen, 1988) based on a pilot study, the power calculation yielded a recommended sample size of at least 20 participants for each group. However, we decided to recruit a larger sample size as recommended in online experiments to reduce variability (Chetverikov & Upravitelev, 2016).

Seventy‐six undergraduate students^1^ (52 females; 10 left‐handed; *M* = 20.2 years; SD = 2.5) from the University of Bologna participated as volunteers. Thirty‐five of them were tested in July 2020, while 41 in January 2021. However, for each group, 28 participants were included in the analysis (for details, see Section 3). All were naïve as to the purpose of the experiment. The study was conducted in accordance with the ethical standards laid down in the Declaration of Helsinki, and fulfilled the ethical standard procedure recommended by the Italian Association of Psychology (AIP). Written consent was obtained from all of them.

### 2.2 Apparatus and stimuli

To create and host the experiment, the online behavioral science platform Gorilla was employed (www.gorilla.sc; Anwyl‐Irvine et al., 2020), already used in previous studies (i.e., Jasmin et al., 2021; Love & Robinson, 2020). In order to minimize possible distractions, participants were invited to carry out the experiment in a quiet place and to avoid manipulating objects during the entire task. In addition, they were asked to close other background apps/programs and all browser windows except for that of the experiment. The automated procedure ensured that participants were all using computers, since no other device was allowed (e.g., tablet, smartphone).

**FIGURE 1:**
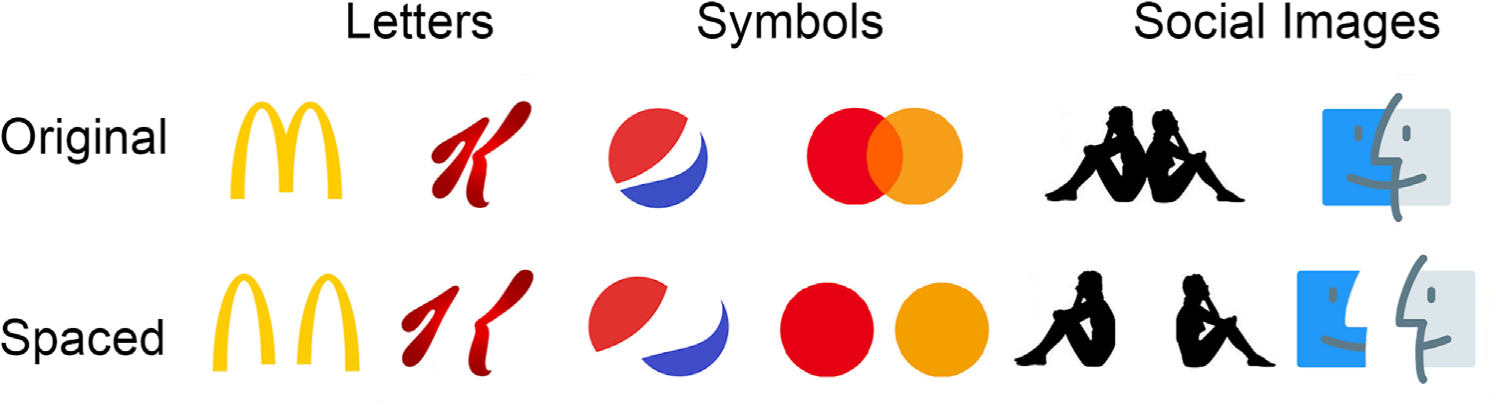
Brand logos, divided by categories (letters, symbols, and social images), in their original (panel above) and spaced (panel below) version

Six brand logos were selected as stimuli on the basis of what they represented: letters (MacDonald's, Kellogg's), symbols (Mastercard, Pepsi), and social images (Finder, Robe di Kappa). Adobe Photoshop software was used to modify the stimuli: each logo was rendered the same area (min 42460 pixel, max 44100 pixel), respecting their original proportions. In addition, they were spaced separating the two halves on the horizontal axis (occupied area by the spaced logos: min 57120 pixel, max 61740 pixel). Each of the 12 logos (6 original and 6 spaced, see Figure 1) was further manipulated to generate a small version (80% of reduction from the starting size) and a large version (120% of increase from the starting size). The final set consisted of 24 stimuli: 6 logos (MacDonald's, Kellogg's, Mastercard, Pepsi, Finder, Robe di Kappa) × 2 size (small, large) × 2 distancing (original, spaced).

### 2.3 Procedure

Each trial began with the presentation of a black fixation cross in the center of a white screen (1000 ms), followed by a central stimulus that remained visible until a response was provided (or for a maximum of 2000 ms). Subsequently, a blank was presented (500 ms). Participants were instructed to maintain the fixation at the center of the screen and to respond according to the size of the logos by using their left and right index fingers. They were instructed to press a left key if the logo was small and a right key if the logo was large (respectively the “e” and “o” keys of a QWERTY keyboard without the numeric keypad; and the “y” and “p” keys of a QWERTY keyboard with the numeric keypad).^2^ Together with instruction, they were given a sample logo (different from the experimental one) with a small and large size to understand the task. Instruction emphasized both speed and accuracy of responses.

Each stimulus was repeated 10 times, thus a total of 240 trials were presented pseudo‐randomly (i.e., random order controlled within participants) across two blocks of 120 trials each. A short rest was allowed between blocks. The experimental section was preceded by a practice session of 12 trials.

At the end of the experimental phase a list with the names of 18 brands (6 from the study plus 12 additional logo names) was presented to verify the effective recognition of the logo and its associated brand, with the purpose of estimating the familiarity of the stimuli. All the additional logos belonged to the same categories as the ones used in the study (i.e., letters, symbols, social images). Participants were required to identify at least 4 out of 6 brands presented in the experimental phase to be included in the analysis.

## 3 RESULTS

Response times (RTs) that were 2 SD faster or slower than each participant's mean (11.9% and 13.7% of the total trials for the 2020 sample and the 2021 sample, respectively) and errors (4.5% and 4.8% of the total trials for the 2020 sample and the 2021 sample, respectively) were excluded from the analysis on RTs. The data are available at: https://osf.io/5p4au/?view_only = 889d10335c404790aa07a2ff5ce29d99.

Seven participants were excluded from the 2020 sample: Four reported a total number of discarded trials (including faster or slower RT and errors) that exceeded the threshold of 1 SD from the overall mean of discarded trials (29.23%)^3^; two participants identified less than four of the six brands (66.6%) presented in the experimental phase; one participant did not complete the experiment within a single session. Therefore, the analysis was conducted on 28 participants (18 females; 10 males; 2 left‐handed; *M* = 20.1 years; SD = 1.3).

Thirteen participants were excluded from the 2021 sample: six reported a total number of discarded trials (including faster or slower RT and errors) that exceeded the threshold of 1 SD from the overall mean of discarded trials (33.4%); six participants identified less than four of the six brands (66.6%) presented in the experimental phase; one launched the experiment without actually doing it. Therefore, the analysis was conducted on 28 participants (18 females; 9 males; 7 left‐handed; *M* = 20.9 years; SD = 3.7).

A repeated‐measures ANOVA was conducted on RTs with *Logos* (MacDonald's, Kellogg's, Mastercard, Pepsi, Finder, Robe di Kappa) and *Distancing* (original vs. spaced) as the within‐subjects factors, and *Sample* (2020 vs. 2021) as the between‐subjects factor, with Huynh–Feldt correction when appropriate. The partial eta‐squared statistic (*η* ^2^_p_), indicating the proportion between the variance explained by one experimental factor and the total variance, was calculated and reported. The *distancing effect* was computed by subtracting the mean RTs for spaced trials from that for the original trials. When necessary, paired sample *t*‐tests were performed.

The analysis revealed a significant main effect of *Logo* (*F*(5,270) = 4.452, *MSE *= 3018.595, *p* = .001, *η* ^2^_p_ = .076), indicating significant slower RTs for Kappa (*M* = 588 ms) compared to all other logos: Kellogg's (*M* = 573 ms, *p* < .001), Mastercard (*M* = 576 ms, *p* < .001), MacDonald's (*M* = 578 ms, *p* = .015), Pepsi (*M* = 578 ms, *p* = .002), and Finder (*M* = 580 ms, *p* = .006); in addition Finder was significantly slower than Kellogg's (*p *= . 044). There was also a main effect of *Distancing* (*F*(1, 54) = 10.328, *MSE *= 6473.853, *p *= .002, *η* ^2^_p_^ ^= .161), showing slower RTs for spaced (*M* = 582 ms) compared to original (*M* = 576 ms) trials; thus an overall *distancing effect* of 6 ms was observed. The main effect of *Sample* was not significant (*F* < 1); however, *Sample* interacted significantly with *Distancing* (*F*(1, 54) = 5.453, *MSE *= 3418.296, *p* = .023, *η* ^2^_p_^ ^= .092), indicating that spaced trials were slower than original trials for the 2021 sample (*M* = 568 ms; *M* = 579 ms, respectively; *distancing effect*: 11 ms, *t*(27) = 3.748, *p *= .001) but not for the 2020 sample (*M* = 583 ms; *M* = 585 ms, respectively; *distancing effect*: 2 ms, *t*(27) = 0.653, *p *= .519).

**FIGURE 2:**
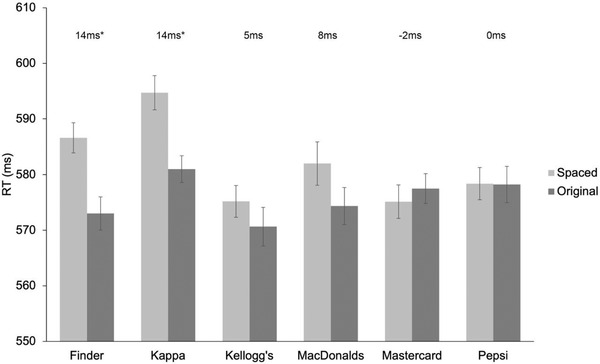
Mean response time (RT, ms) for *Logos* as a function of *Distancing*. Error bars indicate standard errors of the mean adjusted for within‐participants designs (Loftus & Masson, 1994). The magnitude of the *distancing effect*, for each Logo, is reported on top of bars. Asterisks denote significant effects (*p < .005)

Importantly, there was a significant interaction between *Logo* and *Distancing* (*F*(5, 270) = 2.507, *MSE *= 1263.367, *p* = .031, *η* ^2^_p_^ ^= .044), showing that participants were slower with spaced trials compared to original trials only with specific logos. In particular, paired sample *t*‐test revealed that significant differences between original and spaced trials emerged for logos representing social images, that are Finder (*M* = 573 ms; *M* = 587 ms, for original and spaced trials, respectively; *distancing effect*: 14 ms, *t*(55) = 3.338, *p *= .002) and Kappa (*M* = 581 ms; *M* = 595 ms, for original and spaced trials, respectively; *distancing effect*: 14 ms, *t*(55) = 3.215, *p *= .002). No *distancing effects* were observed for the remaining logos (Kellogg's, MacDonald's, Mastercard, and Pepsi). See Figure 2.

No other main effects or interactions were significant (*F_s_ *< 1).

## 4 DISCUSSION

During the COVID‐19 pandemic, to reduce the risk of contagion, isolation and social distancing have been recommended. These acquired behaviors have largely impacted our daily life, mostly on the social dimension. Here, we focus on how spaced logos are processed in the pandemic context, in which social distancing has been imposed. Two groups of participants, recruited in two different moments of the pandemic spread (July 2020 and January 2021), were presented with original and spaced logos showing letters, symbols, and social images. They were asked to discriminate the size of each logo (i.e., large or small).

In line with our hypothesis, results showed a *distancing effect*, that is, participants were slower to process spaced compared to original logos. This result is in line with previous research showing that familiarity facilitates the recognition process of a large variety of stimuli (Konkle & Oliva, 2012; Wang et al., 1994), including brand logos (Qin et al., 2014) supporting the hypothesis that our object recognition system automatically activate stored information (i.e., Grill‐Spector & Kanwisher, 2005), reacting faster to stimuli we are familiar with (i.e., original logos), compared to unfamiliar ones (i.e., spaced logos). It should be noted that during COVID‐19 pandemic, severe attentional failures have been reported (see for example Santangelo et al., 2021). In particular, recent studies have shown that the stressful conditions experienced during the quarantine had an influence on spatial cognition abilities (Lardone et al., 2021; Somma et al., 2021). Specifically, Somma et al. (2021) tested executive functions in a group of students before and during the lockdown and showed a significantly leftward bias (i.e., hyperactivity of right‐hemisphere attention networks that push spatial attention leftward; Toba et al., 2011) in the lockdown period, suggesting more pseudoneglect when behavior is constrained and confirming the correlation between social isolation and the worsening of cognitive functioning. Lardone et al. (2021) extended these results to graphic fluency abilities considered as a spatial competence.

In the light of the above studies, the *distancing effect* reported here could have emerged as an outcome of attentional and visuospatial difficulties raised during the quarantine. Further studies are needed to investigate this aspect.

Interestingly, the *distancing effect* was modulated by both the sample and the type of logo. As for the *Sample*, the *distancing effect* emerged only for participants tested in January 2021, and not for those tested in June/July 2020. At first glance, this result might seem counterintuitive as a habituation effect might be expected: people who followed social distancing practices for a longer time may have integrated them better in their routines than people who followed them for a shorter time. However, it should be considered that the restrictive measures of social distancing act on a phenomenon deeply rooted in human behavior, that is, social interaction (Levinson & Enfield, 2006) and we may assume that the more a phenomenon is rooted in our behavior, the less people may be willing to accept a long‐lasting change concerning it. This would explain why participants from the 2021 sample showed a *distancing effect* whereas participants from the 2020 sample did not.

As for the Logo, results showed that the *distancing effect* emerged for logos representing social images (i.e., Kappa and Finder) compared to other types of logos (i.e., Kellogg's and McDonald's; Mastercard and Pepsi; letters and symbols, respectively). Thus, only logos with a social dimension showed a difficulty to process the spaced version compared to the original one. This disadvantage might reflect the tendency to process physical distancing slowly when human figures are involved. This result suggests that participants project the social distancing experience only on logos that evoke a social and interactional dimension, that is, the dimension mostly affected by measures adopted during the pandemic spread (see for example Peterson et al., 2021; Xu & Cheng, 2021). Indeed, it is likely that measures to contain the pandemic spread have required a greater attentional effort to monitor social contexts on the basis of proximity and distance between people rather than between physical objects.

More importantly, it has been shown that individuals experiencing social isolation or social distancing can develop unpleasant experiences (i.e., Brooks et al., 2020; Peterson et al., 2021). Specifically, in an extensive review, Brooks et al. (2020) describe the psychological impact of quarantine in different epidemia on mental health and psychological wellbeing. Most studies reported negative psychological effects including posttraumatic stress symptoms, confusion, and anger. Examining the impact of social distancing on psychological health and physical activity, Peterson et al. (2021) reported that social distancing has a negative effect on depression, anxiety, and mood. In the light of these negative effects associated with the pandemic context, it could be assumed that social distancing is perceived negatively by the population. Therefore, it is reasonable to assume that the spaced social logos used in this study could have resulted in assuming a negative value, thus determining a resistance in processing them, which results in longer RT. Valence could, therefore, have influenced the processing of the different types of stimuli, as widely demonstrated in the literature, by creating a “negativity bias” that led to slower processing for negative compared to positive stimuli (i.e., word, Bayer & Schacht, 2014; faces, Leppänen & Hietanen, 2004), thus leading to a facilitation for original social logos (i.e., positive stimuli) and an interference for spaced social logos (i.e., negative stimuli).

To conclude, the present study suggests that the acquired distancing behaviors imposed by the pandemic spread affected our performance on brand logos processing, especially when, through spaced social logos, distance in social interaction is evoked. This is the dimension mostly affected by the restrictive measures applied during the pandemic, and being social interaction deeply rooted in human behavior (Ciardo et al., 2015; Levinson & Enfield, 2006), a stronger effect emerges in participants who have been exposed longer to restrictive measures of social distancing.

These findings highlight the novelty of our study, demonstrating how social distancing can have potential repercussions on the perceptual processing of stimuli in the environment that surrounds us. Importantly, the present study shows the role of the wide pandemic contest on perception, two phenomena that apparently are not related to suppose any influence of the first on the second, but that are somehow connected.

To make our provisional conclusion stronger, further studies will proceed with a stronger validation of the logos classification and with a larger number of stimuli.

## FUNDING

This research was not supported by any funding.

### PEER REVIEW

The peer review history for this article is available at https://publons.com/publon/10.1002/brb3.2501

## Data Availability

The data are available at: The data are available at: https://osf.io/5p4au/?view_only=889d10335c404790aa07a2ff5ce29d99

## ENDNOTES

1 These data have been already partially published as a brief report in the Italian journal *Giornale Italiano di Psicologia* (D'Ascenzo et al., in press).

2 Please note that response code was not balanced among participants to avoid any possible congruency effect between response position and size. Several studies indeed demonstrated that information related to object size is automatically spatially coded: small stimuli are represented on the left and large stimuli on the right (see for example Walsh, 2003).

3 For both samples, since the experiment was conducted online, we decided to avoid including in the analysis participants with a high number of discarded trials for both errors and faster or slower response times.
